# Age-related trends in eating pathology symptoms among transgender and gender-diverse adults

**DOI:** 10.1007/s40519-025-01779-4

**Published:** 2025-08-13

**Authors:** Jason M. Nagata, Christopher D. Otmar, Christopher M. Lee, Emilio J. Compte, Jason M. Lavender, Tiffany A. Brown, Kelsie T. Forbush, Annesa Flentje, Micah E. Lubensky, Juno Obedin-Maliver, Mitchell R. Lunn

**Affiliations:** 1 Department of Pediatrics, University of California, San Francisco, 550 16th Street, 4th Floor, Box 0503, San Francisco, CA 94143 USA; 2 Eating Behavior Research Center, School of Psychology, Universidad Adolfo Ibáñez, Santiago, Chile; 3 Research Department, Comenzar de Nuevo Treatment Center, Monterrey, México; 4 Military Cardiovascular Outcomes Research Program (MiCOR), Department of Medicine, Uniformed Services University of the Health Sciences, Bethesda, MD USA; 5 The Metis Foundation, San Antonio, TX USA; 6 Department of Psychological Sciences, Auburn University, Auburn, AL USA; 7 Department of Clinical Child Psychology, University of Kansas, Lawrence, KS USA; 8 The PRIDE Study/PRIDEnet, Stanford University School of Medicine, Stanford, CA USA; 9 Stanford Prevention Research Center, Department of Medicine, School of Medicine, Stanford University, Stanford, CA USA; 10 Alliance Health Project, Department of Psychiatry and Behavioral Sciences, University of California, San Francisco, San Francisco, CA USA; 11 Department of Obstetrics and Gynecology, Stanford University School of Medicine, Stanford, CA USA; 12 Division of Nephrology, Department of Medicine, Stanford University School of Medicine, Stanford, CA USA; 13 Department of Epidemiology and Population Health, Stanford University School of Medicine, Stanford, CA USA

**Keywords:** Eating disorders, Transgender, Lifespan, Age, Gender-diverse, LGBTQIA +

## Abstract

**Purpose:**

This study examined how eating disorder symptoms, assessed by the Eating Pathology Symptoms Inventory (EPSI), vary across chronological age in a large national (USA) sample of transgender and gender-diverse (TGD) adults.

**Method:**

Participants were 2098 TGD adults—including transgender men (*n* = 599), transgender women (*n* = 293), and gender-diverse individuals (*n* = 1,206, including nonbinary and those who identified with “another gender identity”)—enrolled in The PRIDE Study. A multivariate general linear model tested the effects of chronological age, gender group (with gender-diverse as the reference), and their interaction on the eight EPSI scales.

**Results:**

Multivariate analyses showed significant main effects of age (*V* = .045, p < .001) and gender group (*V* = .098, p < .001), but no significant age-by-group interaction. Older age was associated with greater Cognitive Restraint (*β* = .22, p < .001), Negative Attitudes toward Obesity (*β* = .22, p < .001), and Excessive Exercise (*β* = .12, p = .001). Compared to gender-diverse individuals, transgender men exhibited higher Muscle Building, Cognitive Restraint, and Excessive Exercise scores, whereas transgender women reported higher Binge Eating, Purging, Cognitive Restraint, and Negative Attitudes toward Obesity, but lower Muscle Building. A single significant interaction indicated that transgender women showed stronger age-related differences in Purging.

**Conclusions:**

These findings contribute to growing evidence that disordered eating symptoms may not simply resolve with age among TGD individuals and necessitate lifespan-sensitive approaches to eating disorder care. These patterns likely capture a mix of aging processes and cohort-specific exposures to weight-normative and cis-normative ideals.

*Level of evidence* Level V: based on descriptive studies.

**Supplementary Information:**

The online version contains supplementary material available at 10.1007/s40519-025-01779-4.

## Introduction

Eating disorders represent a significant public health concern among transgender and gender-diverse (TGD) individuals [1]. Although elevated rates of disordered eating have been documented across many populations [2], TGD adults face markedly higher risk [3], with lifetime prevalence estimates of eating disorders reaching 10.5% among transgender men and 8.1% among transgender women [4]. Rates of anorexia nervosa and bulimia nervosa are similarly elevated, affecting 4–5% of transgender adults—substantially higher than rates observed among cisgender peers [4]. Research increasingly points to the cumulative effects of structural stigma, systemic discrimination, and body image distress tied to marginalized gender identity as key contributors to this disparity [5–7]. For many TGD individuals, disordered eating behaviors may function as strategies for managing body dysphoria or mitigating distress linked to societal expectations around gendered embodiment [1, 8].

Although these eating disorder disparities among TGD populations are well established, much less is known about how symptoms unfold across different stages of adulthood. This gap underscores a broader limitation in the literature, wherein most studies have focused on youth and early adulthood [9]; this likely reflects a longstanding assumption that eating disorders naturally decline with age [10]. However, emerging meta-analytic evidence suggests that disordered eating symptoms often persist—and may even shift—across adulthood [11, 12] and may vary across stages of identity development or gender transition [13–15]. Chronic exposure to minority stressors, compounded over time, may contribute to sustained or evolving patterns of disordered eating across adulthood [16, 17]. Although adolescence is often framed as the primary window for vulnerability [18], adulthood introduces new stressors—such as healthcare discrimination and economic marginalization [19]—that may reactivate or reshape disordered eating risk, particularly among those navigating multiple marginalized identities [20, 21]. For example, adult concerns about romantic or sexual partnership, particularly around bodily desirability, disclosure, and gender affirmation in intimate contexts, may heighten appearance monitoring. These concerns can intensify weight-shape control behaviors, especially among individuals navigating dating or relational uncertainty post-transition [22–24]. Understanding how eating disorder symptoms are patterned across age is therefore critical to capture the full scope of health inequities faced by TGD communities.

The Eating Pathology Symptoms Inventory (EPSI) [25] offers a multidimensional assessment of eating disorder symptoms and has been increasingly adopted in research with TGD populations. Although originally developed using cisgender samples, recent work demonstrates that the EPSI performs reliably among TGD individuals, with strong support for its factor structure and measurement invariance across TGD populations [26]. Nagata et al. (2025) [27] also established community norms for interpreting EPSI scores among TGD adults, which provide population-specific benchmarks for understanding symptom severity and variation. The EPSI was selected over legacy measures such as the Eating Disorder Inventory (EDI) due to its broader construct coverage [25], evidenced psychometric performance [26, 28], and greater conceptual alignment with the gendered body concerns often reported by TGD individuals. These advances have created critical tools for assessing disordered eating in TGD communities. However, important questions remain about how symptom patterns may differ across stages of adulthood. The present study extends this work by examining how eating disorder symptoms, assessed using the EPSI, vary across chronological age within a large, national sample of TGD adults.

## Methods

### Procedure and participants

This cross-sectional study leveraged data from The PRIDE Study, an ongoing, national cohort study established to investigate health trajectories among sexual and/or gender minority (SGM) and/or LGBTQIA + (lesbian, gay, bisexual, transgender, queer and/or questioning, intersex, asexual, and those who identify with a sexual orientation or gender identity not covered in the acronym) adults (18 years of age or older) residing in the United States and its territories who could independently complete online surveys in English. Data collection was conducted remotely using a secure, cloud-based platform to maximize accessibility. Recruitment strategies included digital engagement through PRIDEnet, targeted social media campaigns, community event outreach, and participant-driven referrals. Full details regarding cohort design, recruitment, and retention have been published previously [29, 30]. The analytic sample for the present study was drawn from the ‘Eating and Body Image 2023’ survey, an ancillary data collection effort conducted between July 2023 and January 2024. Of the 4,729 participants who completed the survey, 2,098 individuals who identified as transgender or gender-diverse (TGD) were retained for analysis. Respondents who completed the survey were eligible to enter a raffle for one of fifty $40 gift cards. All study procedures were approved by the Institutional Review Boards of Stanford University (#63,400), the University of California, San Francisco, and the WIRB-Copernicus Group.

Among transgender men, the mean age was 33.6 years (SD = 11.6, range = 18–83). A total of 91.82% identified as White, including those who selected White alone or in combination with another racial or ethnic identity; 10.68% identified with more than one racial or ethnic group. Transgender women had a mean age of 46.5 years (SD = 15.2, range = 19–86), with 90.44% identifying as White (alone or in combination), and 9.56% identifying with multiple racial/ethnic groups. Participants with gender diversity had a mean age of 33.5 years (SD = 10.5, range = 18–81), with 89.72% identifying as White (alone or in combination), and 14.42% identifying with multiple racial/ethnic groups. Across all groups, the majority of participants had completed a college degree or higher (see Supplemental Table S1 for participant sociodemographic information). Missing data were minimal (< 1% per item) [31].

### Measures

#### Gender identity

Gender identity was assessed via a single self-report item asking participants to select the term that best described their current identity. Participants who selected “transgender man” (n = 599), “transgender woman” (n = 293), “non-binary,” or “another gender identity” were eligible for inclusion. Individuals who selected “cisgender man,” “cisgender woman,” or declined to respond were excluded from analyses. Consistent with recent terminology guidance [32, 33], the latter two categories (“non-binary” and “another gender identity”) were combined into an inclusive gender-diverse group (n = 1,206). This reflects identities that do not align with binary transgender categories. Free-text responses under “another gender identity” (e.g., “gender-fluid,” “transmasculine”) were examined and retained within the broader gender-diverse group due to small cell sizes. As a sensitivity check, no significant differences were found between the non-binary and “another gender identity” subgroups on any EPSI subscale.

#### Eating pathology symptoms inventory (EPSI)

Eating pathology symptoms were measured using the Eating Pathology Symptoms Inventory (EPSI) [25], a 45-item self-report measure comprising eight subscales: Body Dissatisfaction, Binge Eating, Cognitive Restraint, Purging, Restricting, Excessive Exercise, Muscle Building, and Negative Attitudes toward Obesity. Participants rated the frequency of behaviors over the past four weeks on a five-point Likert scale (0 = “Never” to 4 = “Very Often”), with higher scores indicating greater symptom severity. Internal consistency reliability for the EPSI subscales was acceptable to excellent (αs = 0.75-0.91) in the present sample. EPSI subscales were calculated as sums of item responses, creating count-like composites rather than averaged scores. To place outcomes on a common metric and aid interpretation of regression coefficients, subscale scores were z-standardized prior to modeling. We have previously validated and published norms of the EPSI in TGD [26, 27] and sexual minority [34, 35] adults based on data from The PRIDE Study.

#### Age

Participants reported their chronological age in years. Age was treated as a continuous variable and was mean-centered (*M* = 35.34, *SD* = 12.30, *range* = 18–86) prior to model specification.

### Analytic strategy

Data were screened for missingness, normality, and multicollinearity. Given the low rate of missingness (< 0.01% per item) and the absence of any discernible patterns by variable or group, missing data were judged to be missing completely at random. Generalized variance inflation factors (GVIFs) were computed, with all adjusted GVIFs falling below the recommended threshold of 2 (age = 1.99; group = 1.11; age x group = 1.50). Assumptions of linearity, homoscedasticity, and multivariate normality were assessed through residual diagnostics and were judged to be adequately met.

A multivariate general linear model was conducted with the eight z-scored EPSI subscales entered simultaneously as dependent variables. Centered age, gender group (transgender men, transgender women, gender-diverse), and their interaction (age x group) were entered as predictors. Gender-diverse was used as the reference category for all analyses. Omnibus multivariate effects were tested using Pillai’s trace and type III sum of squares estimation. Statistically significant omnibus effects were followed up with univariate multiple regressions for each EPSI subscale using the same predictors. Benjamini–Hochberg false discovery rate (FDR) corrections were applied to control for Type I error across the 48 univariate model terms. For each regression, unstandardized coefficients (b), standardized coefficients (β), standard errors (SE), t-values, 95% confidence intervals (CIs), and model R^2^ values were reported. All analyses were conducted using R version 4.5.0 [36]. Data cleaning and wrangling were performed using the ‘dplyr’ package (version 1.1.4) [37] and ‘janitor’ (version 2.2.1) [38]. Descriptive statistics, internal consistency (Cronbach’s α), and correlation matrices were computed using ‘psych’ (version 2.4.1) [39], and multivariate linear models with interaction terms were estimated using ‘car’ (version 3.1–3) [40]. Standardized effect sizes were computed with ‘lm.beta’ (version 1.7–2) [41] and false discovery rate corrections were applied using broom (version 1.0.8) [42].

Gender-diverse individuals were selected as the reference group for two primary reasons. First, they represented the largest proportion of the analytic sample, which allowed for greater model precision and interpretability. Second, consistent with an equity-focused framework [43], centering gender-diverse individuals challenges the normative positioning of binary identities as baseline groups. This decision aligns with broader efforts within global health research to promote more inclusive practices by elevating the experiences of historically underrepresented groups [44].

## Results

### Multivariate omnibus test

Descriptive statistics for each EPSI scale by gender identity are provided in Supplemental Table S2. Bivariate correlations among the eight EPSI subscales (Supplemental Table S3) revealed moderate associations. The strongest observed associations were between Cognitive Restraint and Excessive Exercise (*r* = 0.51) and between Body Dissatisfaction and Binge Eating (*r* = 0.43). The multivariate regression model demonstrated a significant main effect of age, *V* = 0.045, F (8, 2085) = 12.40, *p* < 0.001, and a significant main effect of gender group, *V* = 0.098, *F* (16, 4172) = 13.38, *p* < 0.001. The interaction between age and gender group was not statistically significant, *V* = 0.010, *F* (16, 4172) = 1.28, *p* = 0.201.

### Follow-up univariate regressions

**Fig. 1:**
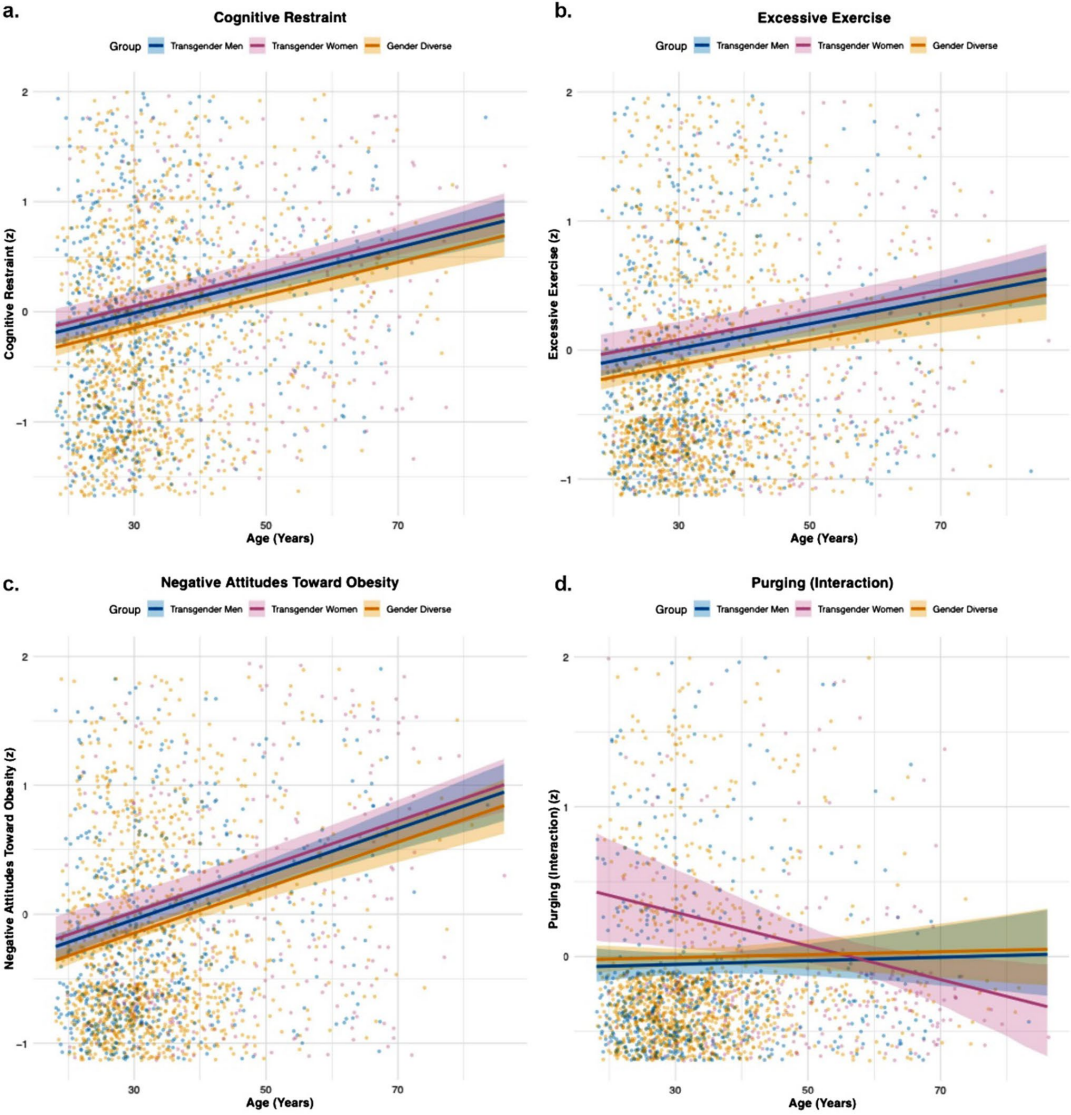
**a**. Positive association between age and cognitive restraint by gender identity. **b**. Positive association between age and excessive exercise by gender identity. **c**. Positive association between age and negative attitudes toward obesity by gender identity. **d**. Age × gender interaction predicting purging symptoms. A significant interaction was observed such that transgender women reported sharper age-related declines in purging symptoms relative to gender-diverse individuals

Follow-up univariate regressions were conducted for each EPSI subscale. Across the 48 regression coefficients, p-values were adjusted using the Benjamini–Hochberg FDR procedure, with significance determined at an adjusted α of 0.05. Complete regression results are presented in Table 1. As shown in Fig. 1(a-c), significant positive age effects were observed for Cognitive Restraint (β = 0.22, b = 0.02, 95% CI [0.01, 0.02], p < 0.001), Negative Attitudes Toward Obesity (β = 0.22, b = 0.02, 95% CI [0.01, 0.02], p < 0.001), and Excessive Exercise (β = 0.12, b = 0.01, 95% CI [0.00, 0.01], p = 0.001). Compared to gender-diverse individuals, transgender men demonstrated higher scores on Muscle Building (β = 0.20, b = 0.43, 95% CI [0.34, 0.53], p < 0.001), Cognitive Restraint (β = 0.06, b = 0.13, 95% CI [0.03, 0.23], p = 0.037), and Excessive Exercise (β = 0.06, b = 0.12, 95% CI [0.03, 0.22], p = 0.050). Transgender women exhibited higher scores than participants with gender diversity on Binge Eating (β = 0.08, b = 0.24, 95% CI [0.09, 0.39], p = 0.011), Purging (β = 0.08, b = 0.24, 95% CI [0.09, 0.39], p = 0.011), Cognitive Restraint (β = 0.08, b = 0.26, 95% CI [0.11, 0.41], p = 0.005), and Negative Attitudes Toward Obesity (β = 0.06, b = 0.18, 95% CI [0.03, 0.33], p = 0.056). In contrast, transgender women reported significantly lower scores on Muscle Building compared to gender-diverse individuals (β = -0.15, b = −0.42, 95% CI [−0.57, −0.27], p < 0.001). A single interaction effect emerged in which the association between age and transgender woman status predicted steeper declines in purging scores relative to gender-diverse individuals (β = -0.08, b = -0.01, 95% CI [−0.02, 0.00], p = 0.038) (see Fig. 1d). No other interaction terms were statistically significant following FDR adjustment. Model-level R^2^ values ranged from 0.00 to 0.07 across EPSI domains, indicating small but interpretable proportions of explained variance. Supplemental Table S4 presents results from models adjusted for educational attainment, income and race/ethnicity that showed similar patterns of association.

**Table 1:** Multivariate regression predicting EPSI scales by age and gender identity

Outcome	Predictor	b	*β*	SE	t	*p*	95% CI	*R*^*2*^
Body Dissatisfaction	(Intercept)	− 0.03		0.03	− 1.08	0.408	[− 0.09, 0.03]	0.00
	Age	0.00	0.04	0.00	1.15	0.408	[0.00, 0.01]	
	Transgender Men	0.10	0.05	0.05	2.02	0.263	[0.00, 0.20]	
	Transgender Women	0.07	0.03	0.08	0.94	0.408	[− 0.08, 0.23]	
	Age × Transgender Men	0.00	− 0.02	0.00	− 0.83	0.408	[− 0.01, 0.01]	
	Age × Transgender Women	− 0.01	− 0.04	0.00	− 1.32	0.408	[− 0.02, 0.00]	
Binge Eating	(Intercept)	− 0.06		0.03	− 1.95	0.102	[− 0.11, 0.00]	0.01
	Age	0.00	− 0.01	0.00	− 0.33	0.745	[− 0.01, 0.00]	
	Transgender Men	0.11	0.05	0.05	2.15	0.096	[0.01, 0.21]	
	Transgender Women	**0.24**	**0.08**	**0.08**	**3.11**	**0.011**	**[0.09, 0.39]**	
	Age × Transgender Men	0.00	− 0.01	0.00	− 0.40	0.745	[− 0.01, 0.01]	
	Age × Transgender Women	− 0.01	− 0.04	0.00	− 1.19	0.354	[− 0.01, 0.00]	
Cognitive Restraint	(Intercept)	− 0.06		0.03	− 2.13	0.050	[− 0.12, 0.00]	0.05
	Age	**0.02**	**0.22**	**0.00**	**6.50**	**0.000**	**[0.01, 0.02]**	
	Transgender Men	**0.13**	**0.06**	**0.05**	**2.64**	**0.017**	**[0.03, 0.23]**	
	Transgender Women	**0.26**	**0.09**	**0.08**	**3.38**	**0.002**	**[0.11, 0.41]**	
	Age × Transgender Men	0.00	− 0.01	0.00	− 0.39	0.696	[− 0.01, 0.01]	
	Age × Transgender Women	− 0.01	− 0.06	0.00	− 1.86	0.075	[− 0.02, 0.00]	
Purging	(Intercept)	0.00		0.03	− 0.05	0.963	[− 0.06, 0.06]	0.01
	Age	0.00	0.01	0.00	0.36	0.963	[ 0.00, 0.01]	
	Transgender Men	− 0.04	− 0.02	0.05	− 0.87	0.767	[− 0.14, 0.06]	
	Transgender Women	**0.24**	**0.08**	**0.08**	**3.06**	**0.014**	**[ 0.09, 0.39]**	
	Age × Transgender Men	0.00	0.00	0.00	0.05	0.963	[− 0.01, 0.01]	
	Age × Transgender Women	− **0.01**	− **0.08**	**0.00**	− **2.60**	**0.028**	**[**− **0.02, 0.00]**	
Restricting	(Intercept)	0.00		0.03	− 0.11	0.949	[− 0.06, 0.05]	0.01
	Age	0.00	− 0.06	0.00	− 1.76	0.470	[− 0.01, 0.00]	
	Transgender Men	− 0.02	− 0.01	0.05	− 0.32	0.949	[− 0.12, 0.08]	
	Transgender Women	0.00	0.00	0.08	− 0.06	0.949	[− 0.16, 0.15]	
	Age × Transgender Men	− 0.01	− 0.03	0.00	− 1.16	0.736	[− 0.01, 0.00]	
	Age × Transgender Women	0.00	0.03	0.00	0.79	0.857	[− 0.01, 0.01]	
Excessive Exercise	(Intercept)	− 0.06		0.03	− 2.19	0.043	[− 0.12, − 0.01]	0.02
	Age	**0.01**	**0.12**	**0.00**	**3.47**	**0.003**	**[0.00, 0.01]**	
	Transgender Men	**0.12**	**0.06**	**0.05**	**2.47**	**0.041**	**[0.03, 0.22]**	
	Transgender Women	**0.17**	**0.06**	**0.08**	**2.24**	**0.043**	**[0.02, 0.32]**	
	Age × Transgender Men	0.00	− 0.01	0.00	− 0.26	0.792	[− 0.01, 0.01]	
	Age × Transgender Women	0.00	0.02	0.00	0.47	0.764	[− 0.01, 0.01]	
Negative Attitudes Toward Obesity	(Intercept)	− 0.05		0.03	− 1.84	0.099	[− 0.11, 0.00]	0.06
	Age	**0.02**	**0.22**	**0.00**	**6.75**	**0.000**	**[0.01, 0.02]**	
	Transgender Men	0.11	0.05	0.05	2.15	0.064	[0.01, 0.20]	
	Transgender Women	0.18	0.06	0.08	2.35	0.056	[0.03, 0.33]	
	Age × Transgender Men	0.00	0.00	0.00	0.01	0.992	[− 0.01, 0.01]	
	Age × Transgender Women	0.00	− 0.01	0.00	− 0.40	0.830	[− 0.01, 0.01]	
Muscle Building	(Intercept)	− 0.06		0.03	− 2.20	0.056	[− 0.12, − 0.01]	0.07
	Age	0.00	0.05	0.00	1.43	0.231	[0.00, 0.01]	
	Transgender Men	**0.43**	**0.20**	**0.05**	**8.90**	**0.000**	**[0.34, 0.53]**	
	Transgender Women	− **0.42**	− **0.15**	**0.08**	− **5.57**	**0.000**	**[**− **0.57, **− **0.27]**	
	Age × Transgender Men	0.00	0.00	0.00	0.04	0.965	[− 0.01, 0.01]	
	Age × Transgender Women	0.00	− 0.02	0.00	− 0.49	0.746	[− 0.01, 0.01]	

Gender-diverse individuals served as the reference group. Coefficients represent unstandardized (b) and standardized (β) estimates. p-values are Benjamini–Hochberg adjusted to control the false discovery rate. Confidence intervals are presented as [lower, upper]

## Discussion

Drawing on a large national sample of over 2,000 TGD adults, the results corroborate and extend evidence that eating disorder risk is not restricted to youth [11, 12, 45]. Multivariate analyses showed that higher age was associated with higher cognitive restraint, stronger anti-obesity attitudes, and more compulsive exercise, which echoes network-analytic work in cisgender samples revealing that weight-shape overvaluation remains a central node even when overt behaviors wane [46]. Conversely, scores for purging and (to a lesser extent) binge eating were lower among older TGD adults—especially among transgender women, which may suggest a developmental shift whereby behaviors stabilize or mitigate with age even as the attitudinal “engine” underlying disordered eating intensifies. This finding aligns with long-term follow-ups documenting durable body-image disturbance and psychological rigidity in anorexia nervosa [47] and in chronic binge-eating trajectories [48, 49]. Prior research suggests that such cognitions can sustain impairment decades after behavioral remission [50]. For TGD communities—in which cis-normative appearance ideals, anti-fat bias, and gender-based stigma remain pervasive—these cognitive vulnerabilities may become further entrenched and function as chronic adaptations to identity-related stress [51]. Scores for restricting and muscle-building showed no age-related attenuation; this demonstrated that not all symptoms vary across ages and challenges the assumption that all eating-disorder risk factors naturally resolve in adulthood among these populations. Taken together, our findings add to the growing research on lifespan and stigma-informed models of disordered eating that move beyond youth-centric frameworks.

Interpretations of these findings must consider several limitations. Foremost, the cross-sectional design precludes inferences about intra-individual developmental change; differences observed across age groups may reflect cohort effects rather than true aging trajectories [52]. Generational factors likely influenced several key findings. For instance, the association between older age and higher negative attitudes toward obesity likely reflects greater generational exposure to rigid weight-normative cultural scripts [53]. Older TGD adults came of age during periods when fatness was more consistently pathologized across public health, media, and medical sectors [54, 55]. This may be linked to anti-fat bias as a normative value [56]. Younger cohorts, while still exposed to pervasive weight stigma messages, may have greater familiarity with body positivity, potentially tempering internalized bias [57, 58]. As Lawrence (2004) notes [59], historical public health narratives increasingly framed health as a matter of individual responsibility by emphasizing behaviors such as calorie restriction and vigorous physical activity as ideal indicators of personal well-being. A similar generational logic may underlie the positive associations between age and cognitive restraint, as well as between age and excessive exercise, observed in the present study. These age differences may reflect historical context rather than developmental change, and there is little evidence that disordered eating symptoms fade over time among TGD adults. Longitudinal designs capable of disentangling cohort effects from true developmental processes are needed in future research, especially within marginalized populations shaped by historical exposures to structural stigma. Moreover, further nuance is needed when considering how gendered societal beauty ideals and embodiment goals shape body image concerns among TGD individuals, leading to differing presentations. According to previous studies, transgender men have a heightened risk of engaging in muscle-building behaviors compared to transgender women and gender-diverse individuals [27, 60]. Conversely, societal norms that idealize thinness in women may lead transgender women to engage in cognitive restraint, excessive exercise, and negative attitudes toward obesity [27, 61].

Additionally, the sample’s racial and socioeconomic composition, predominantly White and highly educated, limits the generalizability of findings to racially and economically marginalized TGD individuals who face additional and distinct structural risk factors for disordered eating. Despite these limitations, the present findings carry significant implications for clinical research and public health. First, there is a need to decouple disordered eating from adolescent-specific frameworks and recognize its persistence—and evolving forms—across the TGD adult lifespan. Screening and intervention efforts should extend into adulthood with particular attention to cognitive restraint, internalized weight stigma, anabolic–androgenic steroid use, social media consumption, and excessive exercise behaviors that may otherwise be overlooked. It is important to understand how these factors contribute to the internalization of unrealistic body ideals. This is especially true for marginalized groups, including LGBTQ + populations, who face additional pressures to conform to heteronormative and cis-normative beauty standards online [62]. Finally, these findings argue for a shift toward lifespan-sensitive models of eating disorder care. Future longitudinal research that integrates measures of minority stress exposure and body image experiences will be essential to building a more complete understanding of eating disorder trajectories in TGD populations.

### Strength and limits

This study leveraged a large, national sample of transgender and gender-diverse adults and a validated multidimensional measure (EPSI) to examine age-related patterns in disordered eating. Limitations include the cross-sectional design, limited racial and socioeconomic diversity, and reliance on self-report.

### What is already known on the subject?

Transgender and gender-diverse individuals are at elevated risk for disordered eating compared to cisgender populations. This is driven in part by identity-related body distress, stigma, and structural discrimination. Prior research has largely focused on adolescents and young adults, with relatively few studies examining how disordered eating symptoms present across the adult lifespan or employing validated, multidimensional symptom inventories in large, community-based samples.

### What this study adds?

Findings from this study indicate that disordered eating symptoms persist across adulthood in transgender and gender-diverse populations and follow specific patterns with age—for example, showing increases in cognitive restraint and internalized weight stigma. By explicitly modeling chronological age, this study demonstrates the importance of developing screening and intervention strategies that are both lifespan-informed and sensitive to the unique experiences of transgender and gender-diverse individuals.

## Supplementary Information

Below is the link to the electronic supplementary material.

Supplementary file 1.

## Data Availability

Data from The PRIDE Study may be accessed through an Ancillary Study application (details at pridestudy.org/collaborate).
