# Supplementary material for: Age-related trends in eating pathology symptoms among transgender and gender-diverse adults
Source: Eat Weight Disord. 2025 Aug 13;30(1):62. doi: 10.1007/s40519-025-01779-4 (PMC12350560; doi:10.1007/s40519-025-01779-4)
Supplement: Supplementary file 1 [file 40519_2025_1779_MOESM1_ESM.docx]

Supplemental Information

Title: Age-related trends in eating pathology symptoms among transgender and gender-diverse adults

Journal: Eating and Weight Disorders - Studies on Anorexia, Bulimia and Obesity

Authors: Jason M. Nagata,^1*^ Christopher D. Otmar,^1*^ Christopher M. Lee,^1^ Emilio J. Compte,^2,3^ Jason M. Lavender,^4,5^ Tiffany A. Brown,^6^ Kelsie T. Forbush,^7^ Annesa Flentje,^9,10,11^ Micah E. Lubensky,^8,9^ Juno Obedin-Maliver,^8,11,13^ Mitchell R. Lunn^8,12, 13^

Affiliations:

^1^ Department of Pediatrics, University of California, San Francisco, San Francisco, CA, USA

^2^ Eating Behavior Research Center, School of Psychology, Universidad Adolfo Ibáñez, Santiago, Chile

^3^ Research Department, Comenzar de Nuevo Treatment Center, Monterrey, México

^4^ Military Cardiovascular Outcomes Research Program (MiCOR), Department of Medicine, Uniformed Services University of the Health Sciences, Bethesda, MD, USA

^5^ The Metis Foundation, San Antonio, TX, USA

^6^ Department of Psychological Sciences, Auburn University, Auburn, AL, USA

^7^ Department of Clinical Child Psychology, University of Kansas, Lawrence, KS, USA

^8^ The PRIDE Study/PRIDEnet, Stanford University School of Medicine, Stanford, CA, USA

^9^ Stanford Prevention Research Center, Department of Medicine, School of Medicine, Stanford University, Stanford, CA, USA

^10^ Alliance Health Project, Department of Psychiatry and Behavioral Sciences, University of California, San Francisco, San Francisco, CA, USA

^11^ Department of Obstetrics and Gynecology, Stanford University School of Medicine, Stanford, CA, USA

^12^ Division of Nephrology, Department of Medicine, Stanford University School of Medicine, Stanford, CA, USA

^13^ Department of Epidemiology and Population Health, Stanford University School of Medicine, Stanford, CA, USA

*Authors contributed equally to the work

Corresponding author email address: [jason.nagata@ucsf.edu](mailto:jason.nagata@ucsf.edu)

| Table S1. Sociodemographic characteristics | | | | | |
| --- | --- | --- | --- | --- | --- |
|  |  | Transgender | Transgender | Gender | p-value |
|  |  | Men | Women | Diverse |  |
| N | | 599 | 293 | 1206 |  |
| Sociodemographic characteristics | | | | | |
| Age | Mean (SD) | 33.5 (11.6) | 46.4 (15.2) | 33.5 (10.4) | <0.001 |
| Ethnicity |  |  |  |  | 0.38 |
|  | American Indian/Alaska Native | 12 (2.00%) | 9 (3.07%) | 49 (4.06%) |  |
|  | Asian | 27 (4.51%) | 14 (4.78%) | 71 (5.89%) |  |
|  | Black/African American | 26 (4.34%) | 11 (3.75%) | 60 (4.98%) |  |
|  | Hispanic/Latino | 38 (6.34%) | 12 (4.10%) | 97 (8.04%) |  |
|  | Middle Eastern/North African | 4 (0.67%) | 3 (1.02%) | 22 (1.82%) |  |
|  | Native Hawaiian/Pacific Islander | 1 (0.17%) | 1 (0.34%) | 3 (0.25%) |  |
|  | White | 550 (91.82%) | 265 (90.44%) | 1082 (89.72%) |  |
|  | Other/Unknown | 5 (0.83%) | 9 (3.07%) | 32 (2.65%) |  |
|  |  |  |  |  |  |
| Sexual orientation | | | |  | <0.001 |
|  | Asexual/Demisexual/Gray-Ace | 60 (10.0%) | 28 (9.6%) | 187 (15.5%) |  |
|  | Bisexual/Pansexual | 181 (30.2%) | 100 (34.1%) | 323 (26.8%) |  |
|  | Gay/Lesbian | 107 (17.9%) | 106 (36.2%) | 180 (14.9%) |  |
|  | Queer | 198 (33.1%) | 43 (14.7%) | 502 (41.6%) |  |
|  | Straight/Heterosexual | 49 (8.2%) | 12 (4.1%) | 6 (0.5%) |  |
|  | Another sexual orientation | 4 (0.7%) | 4 (1.4%) | 8 (0.7%) |  |
| Education | | | |  | <0.001 |
|  | No schooling | 0 (0.0%) | 0 (0.0%) | 1 (0.1%) |  |
|  | Nursery to high school, no diploma | 4 (0.7%) | 1 (0.3%) | 8 (0.7%) |  |
|  | High school graduate or equivalent | 43 (7.2%) | 27 (9.2%) | 73 (6.1%) |  |
|  | Trade/Technical/Vocational training | 14 (2.4%) | 10 (3.4%) | 17 (1.4%) |  |
|  | Some college | 121 (20.3%) | 48 (16.4%) | 188 (15.6%) |  |
|  | 2-year college degree | 37 (6.2%) | 35 (11.9%) | 67 (5.6%) |  |
|  | 4-year college degree | 210 (35.3%) | 84 (28.7%) | 450 (37.3%) |  |
|  | Master’s degree | 113 (19.0%) | 65 (22.2%) | 299 (24.8%) |  |
|  | Doctoral degree | 21 (3.5%) | 10 (3.4%) | 59 (4.9%) |  |
|  | Professional degree | 32 (5.4%) | 13 (4.4%) | 44 (3.6%) |  |
| Income | | | |  | <0.001 |
|  | $0 - $30,000 | 280 (47.4%) | 100 (34.5%) | 554 (46.6%) |  |
|  | $30,001 - $60,000 | 158 (26.7%) | 69 (23.7%) | 329 (27.7%) |  |
|  | $60,001 - $100,000 | 106 (17.8%) | 61 (21.0%) | 195 (16.4%) |  |
|  | $100,001 - $150,000 | 32 (5.4%) | 43 (14.8%) | 78 (6.6%) |  |
|  | $150,001+ | 16 (2.7%) | 18 (6.2%) | 35 (2.9%) |  |
| Note. p-values for continuous variables were calculated using one-way ANOVA, and p-values for categorical variables were derived from Chi-square tests of independence. Participants were allowed to select more than one race/ethnicity, resulting in percentages that exceed 100%. | | | | | |

| Table S2. Descriptive statistics for EPSI scales by gender identity | | | | | | | | | |
| --- | --- | --- | --- | --- | --- | --- | --- | --- | --- |
| **EPSI Subscale** | **α** |  | **M (SD)** | | | | | | |
|  |  |  | *TM* |  | *TW* |  | *GD* |  | *All TGD* |
| Body Dissatisfaction | .88 |  | 14.92 (6.83) |  | 14.45 (5.86) |  | 14.18 (6.81) |  | 14.42 (6.69) |
| Binge Eating | .90 |  | 9.70 (6.94) |  | 10.09 (7.38) |  | 8.94 (6.62) |  | 9.16 (6.77) |
| Cognitive Restraint | .76 |  | 4.38 (3.09) |  | 5.17 (3.24) |  | 3.97 (3.01) |  | 4.26 (3.06) |
| Purging | .78 |  | 0.81 (2.04) |  | 1.17 (2.78) |  | 0.92 (2.36) |  | 0.95 (2.35) |
| Restricting | .87 |  | 6.10 (5.79) |  | 5.98 (5.69) |  | 6.14 (5.61) |  | 6.10 (5.67) |
| Excessive Exercise | .86 |  | 3.78 (4.38) |  | 4.61 (4.92) |  | 3.24 (4.09) |  | 3.58 (4.32) |
| Negative Attitudes | .90 |  | 3.59 (4.29) |  | 4.79 (4.91) |  | 3.15 (3.91) |  | 3.56 (4.25) |
| Muscle Building | .76 |  | 4.44 (4.10) |  | 1.53 (2.67) |  | 2.91 (3.15) |  | 3.16 (3.37) |
| Note. TM = Transgender Men, TW = Transgender Women, GD = Gender Diverse, All TGD = Total transgender and gender diverse sample (N = 2,098). Cronbach’s α indicates internal consistency reliability for each scale in the full analytic sample. | | | | | | | | | |

| Table S3. Bivariate Pearson correlations among EPSI scales | | | | | | | | |
| --- | --- | --- | --- | --- | --- | --- | --- | --- |
|  |  | 1 | 2 | 3 | 4 | 5 | 6 | 7 |
| 1 | Body Dissatisfaction |  |  |  |  |  |  |  |
| 2 | Binge Eating | .43*** |  |  |  |  |  |  |
| 3 | Cognitive Restraint | .43*** | .22*** |  |  |  |  |  |
| 4 | Purging | .36*** | .36*** | .32*** |  |  |  |  |
| 5 | Restricting | .27*** | .06** | .23*** | .24*** |  |  |  |
| 6 | Excessive Exercise | .23*** | .16*** | .51*** | .28*** | .17*** |  |  |
| 7 | Negative Attitudes (Ob.) | .32*** | .30*** | .31*** | .24*** | .12*** | .21*** |  |
| 8 | Muscle Building | .23*** | .13*** | .30*** | .22*** | .15*** | .40*** | .16*** |
| Note: *p* < .001***. Correlations are based on pairwise complete observations. All values are Pearson’s *r*, rounded to two decimal places. | | | | | | | | |

| Table S4. Adjusted multivariate regression models predicting EPSI scales by age and gender identity | | | | | | | | |
| --- | --- | --- | --- | --- | --- | --- | --- | --- |
| **Outcome** | **Predictor** | **b** | ***β*** | **SE** | **t** | ***p*** | **95% CI** | ***R²*** |
| BODY | (Intercept) | .13 |  | .05 | 2.78 | .038 | [0.04, 0.23] | .02 |
|  | Age | .01 | .06 | .00 | 1.78 | .327 | [0.00, 0.01] |  |
|  | Transgender Men | .09 | .04 | .05 | 1.68 | .327 | [-0.01, 0.19] |  |
|  | Transgender Women | .04 | .01 | .08 | 0.49 | .743 | [-0.12, 0.19] |  |
|  | College Degree or Higher | -.22 | -.10 | .05 | -4.39 | .000 | [-0.32, -0.12] |  |
|  | Middle Income ($50K-$100K) | .01 | .00 | .06 | 0.09 | .925 | [-0.10, 0.11] |  |
|  | High Income (>$100K) | -.10 | -.03 | .08 | -1.26 | .462 | [-0.25, 0.05] |  |
|  | Black or African American | .13 | .03 | .11 | 1.14 | .462 | [-0.09, 0.35] |  |
|  | Hispanic/Latino | .09 | .02 | .10 | 0.90 | .573 | [-0.10, 0.27] |  |
|  | Asian | -.08 | -.02 | .10 | -0.80 | .593 | [-0.28, 0.12] |  |
|  | American Indian or Alaska Native | .14 | .02 | .12 | 1.12 | .462 | [-0.10, 0.38] |  |
|  | Multiracial / Other | -.09 | -.01 | .22 | -0.40 | .743 | [-0.53, 0.35] |  |
|  | Age × Transgender Men | .00 | -.01 | .00 | -0.47 | .743 | [-0.01, 0.01] |  |
|  | Age × Transgender Women | -.01 | -.05 | .00 | -1.51 | .367 | [-0.02, 0.00] |  |
| BING | (Intercept) | .08 |  | .05 | 1.62 | .211 | [-0.02, 0.17] | .02 |
|  | Age | .00 | .02 | .00 | 0.49 | .731 | [0.00, 0.01] |  |
|  | Transgender Men | .10 | .05 | .05 | 2.04 | .135 | [0.00, 0.20] |  |
|  | Transgender Women | .22 | .08 | .08 | 2.83 | .033 | [0.07, 0.37] |  |
|  | College Degree or Higher | -.18 | -.08 | .05 | -3.53 | .006 | [-0.28, -0.08] |  |
|  | Middle Income ($50K-$100K) | -.07 | -.03 | .06 | -1.18 | .373 | [-0.17, 0.04] |  |
|  | High Income (>$100K) | -.15 | -.05 | .08 | -1.94 | .135 | [-0.30, 0.00] |  |
|  | Black or African American | .23 | .05 | .11 | 2.09 | .135 | [0.01, 0.45] |  |
|  | Hispanic/Latino | .06 | .01 | .10 | 0.66 | .645 | [-0.12, 0.25] |  |
|  | Asian | .10 | .02 | .10 | 1.00 | .447 | [-0.10, 0.30] |  |
|  | American Indian or Alaska Native | .23 | .04 | .12 | 1.90 | .135 | [-0.01, 0.47] |  |
|  | Multiracial / Other | .00 | .00 | .22 | 0.01 | .989 | [-0.44, 0.44] |  |
|  | Age × Transgender Men | .00 | .00 | .00 | -0.14 | .958 | [-0.01, 0.01] |  |
|  | Age × Transgender Women | -.01 | -.04 | .00 | -1.26 | .366 | [-0.02, 0.00] |  |
| COGN | (Intercept) | -.10 |  | .05 | -2.15 | .090 | [-0.19, -0.01] | .05 |
|  | Age | .02 | .19 | .00 | 5.45 | .000 | [0.01, 0.02] |  |
|  | Transgender Men | .14 | .06 | .05 | 2.88 | .019 | [0.05, 0.24] |  |
|  | Transgender Women | .25 | .09 | .08 | 3.22 | .009 | [0.10, 0.40] |  |
|  | College Degree or Higher | -.02 | -.01 | .05 | -0.35 | .848 | [-0.11, 0.08] |  |
|  | Middle Income ($50K-$100K) | .08 | .04 | .05 | 1.50 | .265 | [-0.02, 0.19] |  |
|  | High Income (>$100K) | .09 | .03 | .08 | 1.15 | .389 | [-0.06, 0.24] |  |
|  | Black or African American | .10 | .02 | .11 | 0.93 | .449 | [-0.11, 0.32] |  |
|  | Hispanic/Latino | .09 | .02 | .09 | 0.96 | .449 | [-0.09, 0.27] |  |
|  | Asian | -.01 | .00 | .10 | -0.12 | .971 | [-0.20, 0.18] |  |
|  | American Indian or Alaska Native | .30 | .05 | .12 | 2.53 | .040 | [0.07, 0.54] |  |
|  | Multiracial / Other | .35 | .03 | .22 | 1.59 | .259 | [-0.08, 0.78] |  |
|  | Age × Transgender Men | .00 | .00 | .00 | 0.00 | .998 | [-0.01, 0.01] |  |
|  | Age × Transgender Women | -.01 | -.04 | .00 | -1.40 | .283 | [-0.02, 0.00] |  |
| PURG | (Intercept) | .05 |  | .05 | 1.11 | .535 | [-0.04, 0.14] | .03 |
|  | Age | .00 | .01 | .00 | 0.30 | .890 | [0.00, 0.01] |  |
|  | Transgender Men | -.05 | -.02 | .05 | -0.95 | .590 | [-0.15, 0.05] |  |
|  | Transgender Women | .21 | .07 | .08 | 2.70 | .024 | [0.06, 0.36] |  |
|  | College Degree or Higher | -.16 | -.08 | .05 | -3.26 | .005 | [-0.26, -0.06] |  |
|  | Middle Income ($50K-$100K) | .00 | .00 | .05 | 0.00 | .997 | [-0.11, 0.11] |  |
|  | High Income (>$100K) | .06 | .02 | .08 | 0.80 | .590 | [-0.09, 0.21] |  |
|  | Black or African American | .50 | .10 | .11 | 4.50 | .000 | [0.28, 0.71] |  |
|  | Hispanic/Latino | .23 | .05 | .09 | 2.41 | .045 | [0.04, 0.41] |  |
|  | Asian | .01 | .00 | .10 | 0.12 | .976 | [-0.18, 0.21] |  |
|  | American Indian or Alaska Native | .48 | .09 | .12 | 3.96 | .001 | [0.24, 0.72] |  |
|  | Multiracial / Other | .18 | .02 | .22 | 0.81 | .590 | [-0.26, 0.61] |  |
|  | Age × Transgender Men | .00 | .01 | .00 | 0.41 | .865 | [-0.01, 0.01] |  |
|  | Age × Transgender Women | -.01 | -.07 | .00 | -2.26 | .056 | [-0.02, 0.00] |  |
| REST | (Intercept) | .31 |  | .05 | 6.77 | .000 | [0.22, 0.40] | .08 |
|  | Age | .00 | -.01 | .00 | -0.23 | .817 | [-0.01, 0.00] |  |
|  | Transgender Men | -.03 | -.01 | .05 | -0.60 | .734 | [-0.13, 0.07] |  |
|  | Transgender Women | -.04 | -.02 | .08 | -0.59 | .734 | [-0.19, 0.10] |  |
|  | College Degree or Higher | -.40 | -.19 | .05 | -8.19 | .000 | [-0.50, -0.30] |  |
|  | Middle Income ($50K-$100K) | -.21 | -.09 | .05 | -3.83 | .000 | [-0.31, -0.10] |  |
|  | High Income (>$100K) | -.25 | -.08 | .07 | -3.37 | .002 | [-0.40, -0.11] |  |
|  | Black or African American | .19 | .04 | .11 | 1.74 | .166 | [-0.02, 0.40] |  |
|  | Hispanic/Latino | .05 | .01 | .09 | 0.52 | .734 | [-0.13, 0.23] |  |
|  | Asian | .14 | .03 | .10 | 1.49 | .237 | [-0.05, 0.33] |  |
|  | American Indian or Alaska Native | .54 | .10 | .12 | 4.56 | .000 | [0.31, 0.77] |  |
|  | Multiracial / Other | .60 | .06 | .22 | 2.77 | .013 | [0.18, 1.03] |  |
|  | Age × Transgender Men | .00 | -.01 | .00 | -0.48 | .734 | [-0.01, 0.01] |  |
|  | Age × Transgender Women | .00 | .01 | .00 | 0.30 | .817 | [-0.01, 0.01] |  |
| EXER | (Intercept) | -.17 |  | .05 | -3.67 | .004 | [-0.27, -0.08] | .04 |
|  | Age | .01 | .07 | .00 | 1.91 | .098 | [0.00, 0.01] |  |
|  | Transgender Men | .14 | .06 | .05 | 2.76 | .018 | [0.04, 0.24] |  |
|  | Transgender Women | .17 | .06 | .08 | 2.15 | .064 | [0.01, 0.32] |  |
|  | College Degree or Higher | .03 | .01 | .05 | 0.52 | .648 | [-0.07, 0.12] |  |
|  | Middle Income ($50K-$100K) | .15 | .07 | .05 | 2.73 | .018 | [0.04, 0.26] |  |
|  | High Income (>$100K) | .22 | .07 | .08 | 2.82 | .018 | [0.07, 0.37] |  |
|  | Black or African American | .09 | .02 | .11 | 0.78 | .553 | [-0.13, 0.30] |  |
|  | Hispanic/Latino | .15 | .03 | .09 | 1.54 | .192 | [-0.04, 0.33] |  |
|  | Asian | -.05 | -.01 | .10 | -0.54 | .648 | [-0.25, 0.14] |  |
|  | American Indian or Alaska Native | .35 | .06 | .12 | 2.88 | .018 | [0.11, 0.59] |  |
|  | Multiracial / Other | .52 | .05 | .22 | 2.34 | .045 | [0.09, 0.96] |  |
|  | Age × Transgender Men | .00 | .00 | .00 | -0.08 | .936 | [-0.01, 0.01] |  |
|  | Age × Transgender Women | .00 | .03 | .00 | 0.85 | .553 | [-0.01, 0.01] |  |
| NATO | (Intercept) | -.01 |  | .05 | -0.26 | .838 | [-0.10, 0.08] | .06 |
|  | Age | .02 | .23 | .00 | 6.76 | .000 | [0.01, 0.02] |  |
|  | Transgender Men | .10 | .05 | .05 | 2.10 | .124 | [0.01, 0.20] |  |
|  | Transgender Women | .17 | .06 | .08 | 2.16 | .124 | [0.02, 0.32] |  |
|  | College Degree or Higher | -.07 | -.04 | .05 | -1.51 | .365 | [-0.17, 0.02] |  |
|  | Middle Income ($50K-$100K) | .01 | .01 | .05 | 0.25 | .838 | [-0.09, 0.12] |  |
|  | High Income (>$100K) | -.05 | -.02 | .08 | -0.71 | .838 | [-0.20, 0.09] |  |
|  | Black or African American | -.02 | .00 | .11 | -0.20 | .838 | [-0.24, 0.19] |  |
|  | Hispanic/Latino | .05 | .01 | .09 | 0.49 | .838 | [-0.14, 0.23] |  |
|  | Asian | .24 | .05 | .10 | 2.46 | .097 | [0.05, 0.43] |  |
|  | American Indian or Alaska Native | .11 | .02 | .12 | 0.94 | .812 | [-0.12, 0.35] |  |
|  | Multiracial / Other | .13 | .01 | .22 | 0.61 | .838 | [-0.30, 0.56] |  |
|  | Age × Transgender Men | .00 | .01 | .00 | 0.27 | .838 | [-0.01, 0.01] |  |
|  | Age × Transgender Women | .00 | -.01 | .00 | -0.33 | .838 | [-0.01, 0.01] |  |
| MUSC | (Intercept) | -.13 |  | .05 | -2.89 | .018 | [-0.22, -0.04] | .08 |
|  | Age | .00 | .03 | .00 | 0.98 | .571 | [0.00, 0.01] |  |
|  | Transgender Men | .44 | .20 | .05 | 9.05 | .000 | [0.35, 0.54] |  |
|  | Transgender Women | -.41 | -.14 | .08 | -5.47 | .000 | [-0.56, -0.27] |  |
|  | College Degree or Higher | .03 | .02 | .05 | 0.68 | .693 | [-0.06, 0.13] |  |
|  | Middle Income ($50K-$100K) | .05 | .02 | .05 | 0.85 | .616 | [-0.06, 0.15] |  |
|  | High Income (>$100K) | .02 | .01 | .07 | 0.28 | .818 | [-0.13, 0.17] |  |
|  | Black or African American | .28 | .06 | .11 | 2.59 | .034 | [0.07, 0.49] |  |
|  | Hispanic/Latino | .11 | .03 | .09 | 1.20 | .463 | [-0.07, 0.29] |  |
|  | Asian | .05 | .01 | .10 | 0.55 | .740 | [-0.14, 0.24] |  |
|  | American Indian or Alaska Native | .19 | .03 | .12 | 1.63 | .291 | [-0.04, 0.42] |  |
|  | Multiracial / Other | .29 | .03 | .22 | 1.35 | .410 | [-0.13, 0.72] |  |
|  | Age × Transgender Men | .00 | .01 | .00 | 0.23 | .818 | [-0.01, 0.01] |  |
|  | Age × Transgender Women | .00 | -.01 | .00 | -0.24 | .818 | [-0.01, 0.01] |  |
| *Note.* Note. Models adjust for age, gender identity (Transgender Men, Transgender Women, Gender Diverse), educational attainment (college degree or higher vs. less than college degree), household income (low: <$50,000; middle: $50,000–$100,000; high: >$100,000), and race/ethnicity (White [reference], Black or African American, Hispanic/Latino, Asian, American Indian or Alaska Native, Multiracial/Other). Gender Diverse individuals served as the reference group for gender identity comparisons. Outcomes are abbreviated as follows: BODY = Body Dissatisfaction, BING = Binge Eating, COGN = Cognitive Restraint, PURG = Purging, REST = Restricting, EXER = Excessive Exercise, NATO = Negative Attitudes Toward Obesity, MUSC = Muscle Building. Coefficients represent unstandardized (b) and standardized (β) estimates. p-values are Benjamini–Hochberg adjusted to control the false discovery rate. Confidence intervals are presented as [lower, upper]. | | | | | | | | |

**Fig. S1.** Age distribution by gender identity group.

*Note.* Boxplots reflect age distributions among transgender men, transgender women, and gender-diverse participants. Dots represent individual observations.
